# Are Thyroid Hormone and Tumor Cell Proliferation in Human Breast Cancers Positive for HER2 Associated?

**DOI:** 10.1155/2015/765406

**Published:** 2015-01-28

**Authors:** Iordanis Mourouzis, Alexandros Tzovaras, Basil Armonis, Alexandros Ardavanis, Maria Skondra, John Misitzis, Demetrios Pectasides, Constantinos Pantos

**Affiliations:** ^1^Department of Pharmacology, University of Athens, 75 Mikras Asias Avenue, Goudi, 11527 Athens, Greece; ^2^Second Department of Internal Medicine, Hippokration Hospital, School of Medicine, University of Athens, 11527 Athens, Greece; ^3^First Department of Medical Oncology, “Saint Savvas” Anticancer Hospital, 11522 Athens, Greece

## Abstract

*Objective.* This study investigated whether thyroid hormone (TH) levels are correlated to cell proliferation (Ki67), in euthyroid breast cancer patients.* Design and Methods.* 86 newly diagnosed breast cancer patients with estrogen receptor (ER) positive tumors, who referred for surgery, were included in the study.* Results.* FT3, FT4, and TSH were within normal range. No correlation was seen between Ki67 and FT3 (*r* = −0.17, *P* = 0.15), FT4 (*r* = −0.13, *P* = 0.25), or TSH (*r* = −0.10, *P* = 0.39) in all patients studied. However, subgroup analysis showed that, in HER2(+) patients, a negative correlation existed between FT3 levels and Ki67 (*r* = −0.60 and *P* = 0.004) but not between Ki67 and FT4 (*r* = 0.04 and *P* = 0.85) or TSH (*r* = −0.23 and *P* = 0.30). In HER2(−) patients, there was no significant correlation between Ki67 and FT3 (*r* = −0.06, *P* = 0.67), FT4 (*r* = −0.15, *P* = 0.26), or TSH (*r* = −0.09, *P* = 0.49). Phospho-p44/total p44 ERK levels were found to be increased by 2-fold in HER2(+) versus HER2(−) tumors. No difference was detected in phospho-p42/total p42 ERK levels.* Conclusions.* TH profile is not altered in patients with newly diagnosed breast cancer. However, FT3 levels, even within normal range, are negatively correlated with cell proliferation in HER2(+) breast cancer tumors. This response may be due to the interaction between ERK and TH signaling.

## 1. Introduction

Thyroid hormone (TH) may be critical in the pathogenesis and progression of diseases due to its regulatory role on cell maturation [1]. There is now growing evidence that thyroid signaling (deiodinases, thyroid hormone receptors) may be altered in cancer or stressed cells due to the activation of growth kinase signaling and this response may be of physiological relevance [2–5]. Furthermore, due to tissue changes in thyroid signaling, even subtle changes in TH levels within normal range may alter the response of cancer cells to thyroid hormone [6]. Consistent with this evidence, FT3 levels in euthyroid patients were found to be inversely correlated to cancer mortality [7]. Thus, based on this evidence, we investigated whether TH levels are correlated to cell proliferation and tumor size in euthyroid patients with breast cancer. This issue has not been previously assessed. Almost a century ago, Beatson showed that this particular group of patients may benefit from treatments with thyroid gland extracts [8]. However, since then, the issue relative to possible associations between thyroid hormone and breast cancer has been debated for decades and remains controversial [9–11].

## 2. Methods

### 2.1. Patients

A total of 86 patients with newly diagnosed breast cancer with estrogen receptor (ER) positive breast tumors, who referred for surgery, were included in the study.

Patients who had radiation or chemotherapy administration before surgery, hormone replacement therapy, any kind of previously diagnosed thyroid disease, and chronic kidney failure were not included. These patients were not on therapy with *β*-blocking agents, aspirin, heparin, phenytoin, steroids, or dopamine in the month before or during the study. In addition, they were not given iodine-containing contrast agents in the six months before and during the study. Thyroid hormone levels and serum measurements were performed prior to surgery in all patients.

Clinical history, age, and menopausal status together with tumor characteristics including tumor histological grade, lymphovascular invasion, lymph node stage, metastases, and tumor size were studied.

Consent has been obtained from each patient or subject after full explanation of the purpose and nature of all procedures used. The investigation was approved by the local ethical committee, functioning according to the 3rd edition of the Guidelines on the Practice of Ethical Committees in Medical Research issued by the Royal College of Physicians of London and in accordance with the Declaration of Helsinki (1964).

### 2.2. Histopathological Analysis

Determination of ER, progesterone receptors (PR), HER2, and Ki67 in breast tumor tissues was performed by immunohistochemical staining. The anti-human antibodies used were ER (Clone : 1D5, Dako, dilution 1 : 300), PR (Clone : PgR636, Dako, dilution 1 : 170), HER2 (polyclonal rabbit anti-human GerbB2, Dako, dilution 1 : 400), and KI67 (Clone : MIB1, Dako, dilution 1 : 150). Their detection was made feasible using Bond Refine Polymer Detection kit and Bond Epitope Retrieval Reagents on a Bond-maX fully automated IHC system (Leica Biosystems GmbH company).

For this study, 86 frozen breast tumor tissues were obtained during surgery with informed patient consent. Usage of these tissues complied with the regulations set by our Institutional Review Board (IRB) for research purposes. All the tumors were diagnosed as invasive carcinoma during the diagnostic workup by certified pathologists.

### 2.3. Thyroid Hormone Levels

Serum TSH, free L-thyroxine (FT4), and free 3,5,3′ tri-iodothyronine (FT3) quantitative measurements were performed using electrochemiluminescence immunoassay (ECLIA) method in COBAS 6000 automated analyzer (ROCHE Diagnostics International Ltd.). TSH is expressed in *μ*IU/mL with normal range between 0.270 and 4.2 *μ*IU/mL. FT4 is expressed in ng/mL with normal range between 0.8 and 2 ng/mL and FT3 is expressed in pg/mL with normal range between 1.8 and 4.6 pg/mL.

### 2.4. SDS-PAGE and Immunoblotting Analysis

Tumor samples were homogenized in ice-cold buffer (A) containing 10 mM HEPES (pH: 7.8), 10 mM KCl, 0.1 mM EDTA, 0.1 mM EGTA, 0.5 mM PMSF, 1 mM DTT, and 10 *μ*g/mL leupeptin. 200 *μ*L of 10% IGEPAL was added and samples were left in ice for 30 min. Homogenization was repeated and the homogenate was centrifuged at 1000 g for 5 min, at 4°C; the pellet was discarded, while the supernatant containing the cytosolic-membrane fraction was stored at −80°C. Protein concentrations were determined by the BCA method.

Samples were prepared for sodium dodecyl sulfate polyacrylamide gel electrophoresis (SDS-PAGE) by boiling for 5 min in Laemmli sample buffer containing 5% 2-mercaptoethanol. 30 *μ*g (cytosolic fraction) of total protein was loaded onto 7% or 10% (w/v) acrylamide gels and subjected to SDS-PAGE in a Bio-Rad Mini Protean gel apparatus. For Western blotting, following SDS-PAGE, proteins were transferred electrophoretically to a nitrocellulose membrane (Hybond ECL) at 100 V and 4°C, for 1.5 h using Towbin buffer. After Western blotting, filters were probed with antibodies against total and phosphorylated ERK (p-ERK) (Cell Signalling Technology, dilution 1 : 1000) and total and phosphorylated Akt (p-Akt) (Cell Signalling Technology, dilution 1 : 1000) overnight at 4°C. Filters were incubated with appropriate anti-rabbit (Cell Signaling) HRP secondary antibodies. Immunoreactivity was detected by enhanced chemiluminescence using Lumiglo reagents (New England Biolabs). Chemiluminescence was detected by the digital image analysis system FluorChem HD2 (Alpha Innotech Corporation, 14743 Catalina Street, San Leandro, CA) equipped with a CCD camera and analysis software. Five samples from HER2(+) and ten samples from HER2(−) group were loaded on the same gel. Results were expressed as the ratio of phosphorylated kinase levels to total kinase levels.

### 2.5. Analysis of Data and Statistics

Analysis of qualitative measurements between groups was performed with *χ* ^2^ test using Pearson equation. Analysis of quantitative measurements between groups was performed using one-way ANOVA. One-way analysis of variance with Bonferroni or Dunnett correction was used for multiple comparisons. Potential correlations between continuous variables were evaluated by the Pearson product-moment (Pearson *r*) or by Spearman's rank correlation coefficient (Spearman *r*), as appropriate. Continuous variables are expressed as mean (SEM), whereas categorical variables are reported as percentages. Significance was set at 0.05. Statistical analysis was performed using the statistical software package SPSS 17.0.

## 3. Results

### 3.1. Patients Baseline Characteristics

Patient age ranged from 27 to 89 years with a mean age of 60.3 (1.6) years. A percentage of 70.9% of patients (61 out of 86) were menopausal (amenorrhea for at least 1 year). A small percentage of 8.1% (7 out of 86) were found to have distant organ metastases at diagnosis. Concerning the tumor histological grade, 9.3% were identified as grade 1, 77.9% as grade 2, and 12.8% as grade 3.

22 patients out of 86 studied were positive for HER2, while 64 were negative for HER2. No significant difference was found in age and clinical stage of the disease between HER2(+) and HER2(−) breast cancer patients (Table 2). Furthermore, the percentage of menopausal women was similar between the two groups. However, the tumor size and the Ki67 index were significantly increased in HER2(+) versus HER2(−) breast cancer patients. No difference was found in the percentage of positive PR tumors between the two groups (Table 2).

### 3.2. Differences in the Activation Pattern of ERK and Akt Signaling between HER2(+) and HER2(−) Breast Tumors

Phospho-p44/total p44 ERK levels were found to be increased by 2-fold in HER2(+) tumors in comparison to HER2(−) tumors. No difference was detected in phospho-p42/total p42 ERK levels between the two groups. Furthermore, a 3.0-fold reduction in phospho-Akt/total Akt was found in HER2(+) in comparison to HER2(−) tumors (Figure 3).

### 3.3. Thyroid Hormone Levels in Breast Cancer Patients

Thyroid hormone levels were shown to be within normal range in ER*α* positive breast cancer patients. No difference in FT3, FT4, and TSH levels was found between premenopausal and postmenopausal patients and between different clinical stages of the disease and histopathological grades (Table 1). No changes in TH levels were seen between HER2 positive and negative patients.

### 3.4. Thyroid Hormone and Tumor Size

No correlation was found between tumor size and FT3 (*r* = −0.12, *P* = 0.3), FT4 (*r* = 0.13, *P* = 0.25), or TSH (*r* = 0.19, *P* = 0.1) when all patients enrolled were included in analysis. Furthermore, subgroup analysis showed that, in HER2(+) breast cancer patients, there was no correlation between tumor size and FT3 (*r* = −0.15 and *P* = 0.54), FT4 (*r* = 0.01 and *P* = 0.99), or TSH (*r* = −0.21 and *P* = 0.37). In HER2(−) breast cancer patients, a weak, borderline correlation was found between tumor size and TSH (*r* = 0.25, *P* = 0.053), but not between tumor size and FT3 (*r* = −0.13, *P* = 0.35) or FT4 (*r* = 0.16, *P* = 0.22).

### 3.5. Thyroid Hormone and Proliferation Index

**Figure 1:**
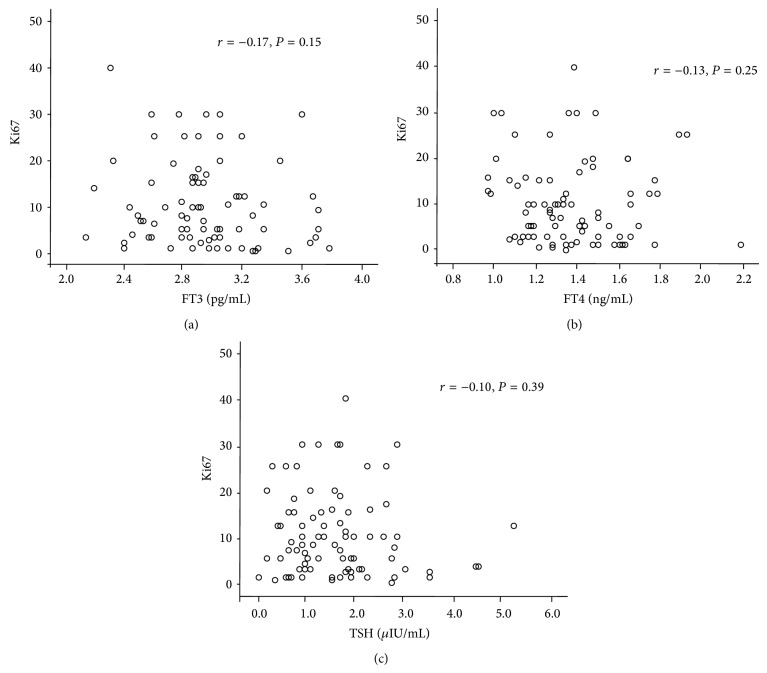
Scatterplots showing no correlation between proliferation index Ki67 and (a) FT3, (b) FT4, and (c) TSH in breast cancer patients including both HER2(−) and HER2(+) tumors.

**Figure 2:**
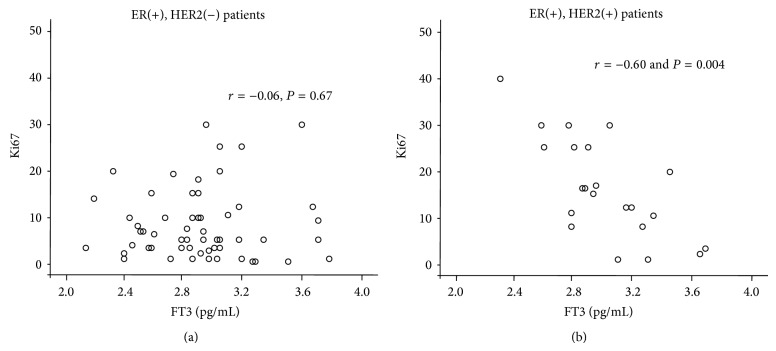
Scatterplots showing correlation between proliferation index Ki67 and FT3 in breast cancer patients including either (a) HER2(−) or (b) HER2(+) tumors. A correlation exists between Ki67 and FT3 only in HER2(+) breast cancer patients.

**Figure 3:**
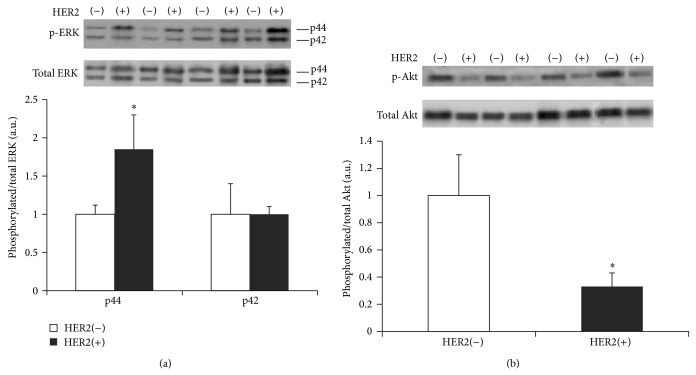
Densitometric assessment and representative Western blots of p44 and p42 phosphorylated ERK/total ERK (a) and phosphorylated Akt/total Akt (b) are shown in breast cancer patients with HER2(−) and HER2(+) tumors. ^*^ *P* < 0.05 versus HER2(−).

No correlation was seen between ki67 and FT3 (*r* = −0.17, *P* = 0.15), FT4 (*r* = −0.13, *P* = 0.25), or TSH (*r* = −0.10, *P* = 0.39) when all patients enrolled were included in analysis (Figure 1). However, subgroup analysis showed that, in HER2(+) breast cancer patients, a negative correlation existed between FT3 levels and Ki67 (*r* = −0.60 and *P* = 0.004) but not between Ki67 and FT4 (*r* = 0.04 and *P* = 0.85) or TSH (*r* = −0.23 and *P* = 0.30) (Figure 2). In HER2(−) breast cancer patients, there was no significant correlation between Ki67 and FT3 (*r* = −0.06, *P* = 0.67), FT4 (*r* = −0.15, *P* = 0.26), or TSH (*r* = −0.09, *P* = 0.49).

## 4. Discussion

This study has provided evidence that changes in thyroid hormone levels within normal range may be associated with proliferative activity of breast tumors in euthyroid patients with breast cancer.

A series of 86 consecutive patients who first presented with ER (estrogen receptor) positive breast cancer and without history of thyroid disorder or TH treatment were included in this study. A high percentage of those women were menopausal. Assessment of TH profile was performed by measuring FT3, FT4, and TSH levels in serum. TH levels in serum were within normal range in all patients with newly diagnosed breast cancer. Furthermore, no differences in TH profile were observed across patients with different clinical stage, histopathological grades, menopause status, and presence of HER2 staining.

In this group of patients, we sought to determine potential correlations of FT3, FT4, and TSH levels with proliferation index (Ki67) and tumor size. FT3, FT4, and TSH were not found to be correlated to either proliferation index or tumor size. However, subgroup analysis revealed a negative correlation between proliferation index (Ki67) and FT3 levels only in HER2 positive patients, while no correlation of FT4 or TSH to proliferation index was observed. Furthermore, tumor size was not correlated to FT3, FT4, and TSH levels in serum. HER2 positive breast cancer patients had comparable characteristics (age, menopausal status, histopathological grade, and clinical stage) to HER2 negative counterparts but greater tumor size and cell tumor proliferation index. These data clearly show that changes of FT3 even within normal range may be of physiological relevance in HER2 positive breast cancer patients. This differential association of HER2 positive and HER2 negative tumors to thyroid hormone is not fully understood. However, overexpression of HER2 receptor can lead to the activation of growth kinase signaling such as ERK [12–14]. Consistent with this evidence, our study showed that ERK activation was greater in HER2 positive tumors, while Akt activation was significantly lower. ERK regulates important downstream target molecules related to extracellular matrix and pathological growth [4, 12]. ERK activation can increase MMP1 which can cause ECM degradation and tumor invasiveness [12]. Furthermore, ERK has been recently shown to interact with the thyroid hormone receptor *α*1, implicating TH signaling in growth development [4]. Thus, T3 treatment of glioma tumor cells, which expressed thyroid hormone receptor *α*1, results in suppressed cell proliferation [6]. This new evidence provides a plausible explanation of our data showing that FT3 levels negatively correlate with cell proliferation in HER2 positive tumors. This issue is of important physiological and clinical relevance and merits further investigation. HER2/ERK signaling is thought to contribute to tamoxifen resistance [14]. Furthermore, newer drugs acting on this pathway appear to induce thyroid dysfunction which may have a detrimental effect [15].

In conclusion, the TH profile is not altered in patients with newly diagnosed breast cancer without concomitant thyroid disease. However, FT3 levels even within normal range are negatively correlated with cell proliferation in HER2 positive breast cancer tumors. This may be of important physiological relevance.

## Figures and Tables

**Table 1:** Comparison of thyroid hormone levels in breast cancer patients based on menopausal status, clinical stage, histopathological grade, and HER2 staining.

	Menopause				Significance
	No	Yes			
FT3	2.97 (0.12)	2.92 (0.05)			*P* = 0.66
FT4	1.39 (0.05)	1.36 (0.03)			*P* = 0.60
TSH	1.67 (0.19)	1.58 (0.13)			*P* = 0.70

	Clinical stage				
	I	II	III	IV	

FT3	2.88 (0.15)	2.95 (0.05)	2.95 (0.13)	2.97 (0.13)	*P* = 0.94
FT4	1.36 (0.06)	1.38 (0.04)	1.36 (0.07)	1.32 (0.08)	*P* = 0.90
TSH	1.35 (0.19)	1.58 (0.14)	2.04 (0.32)	1.85 (0.6)	*P* = 0.27

	Histopathological grade				
	Grade 1	Grade 2	Grade 3		

FT3	2.77 (0.18)	2.95 (0.06)	2.97 (0.14)		*P* = 0.59
FT4	1.26 (0.08)	1.36 (0.03)	1.45 (0.07)		*P* = 0.26
TSH	2.18 (0.67)	1.64 (0.11)	1.21 (0.3)		*P* = 0.16

	HER2 staining				
	Negative	Positive			

FT3	3.0 (0.07)	2.9 (0.06)			*P* = 0.35
FT4	1.36 (0.06)	1.37 (0.03)			*P* = 0.88
TSH	1.74 (0.25)	1.57 (0.12)			*P* = 0.50

**Table 2:** Comparison of clinical and histopathological characteristics in HER2(+) and HER2(−) breast cancer patients.

	HER2 staining		Significance
	Negative (*n* = 64)	Positive (*n* = 22)	
Age (years)	60.3 (1.6)	60.1 (3.3)	*P* = 0.95
Tumor size (cm)	2.3 (0.12)	3.0 (0.28)	**P** = 0.007
Ki67	8 (0.9)	16 (2.3)	**P** = 0.003
Menopause			
No	29.7%	27.3%	*P* = 0.83
Yes	70.3%	72.7%	
Clinical stage			
I	28.1%	13.6%	*P* = 0.40
II	53.1%	54.5%	
III	12.5%	18.2%	
IV	6.3%	13.6%	
Histopathological grade			
1	9.8%	4.5%	*P* = 0.63
2	77%	86.4%	
3	13.2%	9.1%	
PR staining			
Negative	28.6%	33.3%	*P* = 0.68
Positive	71.4%	66.7%	
